# Predicting outcomes in partial nephrectomy: is the renal score useful?

**DOI:** 10.1590/S1677-5538.IBJU.2016.0315

**Published:** 2017

**Authors:** André Costa Matos, Marcos F. Dall´Oglio, José Roberto Colombo, Alexandre Crippa, João A. Q. Juveniz, Felipe Coelho Argolo

**Affiliations:** 1 Hospital São Rafael, Salvador, BA, Brasil;; 2 Departamento de Urologia, Faculdade de Medicina da Universidade de São Paulo, SP, Brasil;; 3 Departamento de Uro-Oncologia,Instituto do Câncer do Estado de São Paulo, SP, Brasil;; 4 Serviço de Urologia, Hospital do Servidor Público Municipal de São Paulo - HSPM, SP, Brasil

**Keywords:** Nephrectomy, Operative Time, Patients

## Abstract

**Introduction and Objective:**

The R.E.N.A.L. nephrometry system (RNS) has been validated in multiple open, laparoscopic and robotic partial nephrectomy series. The aim of this study was to test the accuracy of R.E.N.A.L. nephrometry system in predicting perioperative outcomes in surgical treatment of kidney tumors <7.0cm in a prospective model.

**Materials and Methods:**

Seventy-one patients were selected and included in this prospective study. We evaluate the accuracy of RNS in predicting perioperative outcomes (WIT, OT, EBL, LOS, conversion, complications and surgical margins) in partial nephrectomy using ROC curves, univariate and multivariate analyses. R.E.N.A.L. was divided in 3 groups: low complexity (LC), medium complexity (MC) and high complexity (HC).

**Results:**

No patients in LC group had WIT >20 min, versus 41.4% and 64.3% MC and HC groups respectively (p=0.03); AUC=0.643 (p=0.07). RNS was associated with convertion rate (LC:28.6% ; MC:47.6%; HC:77.3%, p=0.02). Patients with RNS <8 were most often subjected to partial nephrectomy (93% x 72%, p=0.03) and laparoscopic partial nephrectomy (56.8% x 28%, p=0.02), AUC=0.715 (p=0.002). The RNS was also associated with operative time. Patients with a score >8 had 6.06 times greater chance of having a surgery duration >180 min. (p=0.017), AUC=0.63 (p=0.059). R.E.N.A.L. score did not correlate with EBL, complications (Clavien >3), LOS or positive surgical margin.

**Conclusion:**

R.E.N.A.L. score was a good method in predicting surgical access route and type of nephrectomy. Also was associated with OT and WIT, but with weak accuracy. Although, RNS was not associated with Clavien >3, EBL, LOS or positive surgical margin.

## INTRODUCTION

The widespread use of imaging modalities has increased the incidence of renal tumors, which are mostly identified from smaller and incidental renal masses. Thus, at present, more than 60% of such patients are diagnosed with T1 tumors (1). The literature supports that partial nephrectomy (PN) is oncologically similar to total nephrectomy (TN) (2) but is associated with fewer cardiovascular events (3); however, TN remains the most common form of treatment for newly diagnosed small RTs (4, 5). This could be explained by the superior feasibility of TN, especially laparoscopically (6), and the fact that the surgical access decision is subjective for each surgeon based on the tomography exam.

To standardize tumor assessment, minimize bias and improve clinical outcomes, the R.E.N.A.L. nephrometry system (RNS) was proposed in 2009 (7), based on five tumor characteristics (radius, exophytic extent, nearness to the renal sinus, anterior/posterior location and location relative to the polar lines). Since then, this tool has been validated in multiple retrospective open, laparoscopic and robotic partial nephrectomy series (8-12).

However, the authors did not use any statistical model to build the score and their variables won the same weight. We believe that, some anatomical features presented in R.E.N.A.L. score are more important than the others and should, specially in laparoscopic surgery, influence perioperative outcomes.

The aim of this study was to test the accuracy of R.E.N.A.L. score system in predicting perioperative outcomes in laparoscopic partial nephrectomy of kidney tumors ≤7.0cm, in a prospective model.

## MATERIALS AND METHODS

Between January 2010 and June 2012, 320 patients underwent radical or partial nephrectomies at our institution for the treatment of renal cancer. Of these, 173 patients had tumors ≤7cm. Patients with chronic renal failure, solitary kidneys, renal tuberculosis, previous renal or upper abdomen surgeries or nephrolithiasis were excluded. We had also excluded patients without multiplanar CT scan that could disrupt R.E.N.A.L. interpretation. Seventy-one patients were selected and prospectively followed up.

The R.E.N.A.L. score was determined by the same observer based on criteria proposed by Uzzo (7). This system considers tumor size, the degree to which the tumor is endophytic, the proximity to the collecting system, the posterior or anterior location of the mass and its polarized location. Awarded for each component are 1 to 3 points, except for the anterior or posterior location, which receives a letter “A” or “P”. Additionally, a suffix “h” is given to lesions that touch the main artery or vein. Thus, 3 groups were formed, according to tumor complexity: low (LC: patients with score 4-6), medium (MC: patients with score 7-9) and high (HC: patients with score 10-12).

All patients were initially indicated for partial laparoscopic nephrectomy, considered herein as a gold standard. The procedures were performed using the laparoscopic standard technique, briefly described as follows: Mobilization of the colon, dissection of the renal vascular pedicle and removal of the Gerota’s fascia. Warm ischemia was achieved using a vascular clamp. Mannitol was administered 5 min before and after the vascular occlusion. The tumor was located and excised, along with its perinephric fat, using scissors. No frozen section analysis of the tumor bed was routinely performed. Hemostasis and closure of the calices were applied whenever necessary using figure-of-eight 2-0 Vicryl® SH needle sutures (Johnson & Johnson New Brunswick, NJ, USA). An approximation of the renal trauma was performed using 0 Vicryl® CT needle ‘U’ sutures anchored with the Hem-o-lok® Ligation System (Teleflex Incorporated, Limerick, PA USA). No ureteric stent was placed in any case. Patient baseline and tumor characteristics are depicted in Table-1.

**Table 1:** Clinicopathological data and surgical approaches.

Gender	
Male	39 (55%)
Female	32 (45%)
Age	60±12.7 (22-88)
IMC	27.6±4.5 (17.9-88)
ASA	
I	5 (7%)
II	60 (84.5%)
III	6 (8.5%)
Hypertension	32 (45.1%)
DM	9 (12.7%)
Smokers	19 (26.8%)
Charlson	
≤3	40 (56.3%)
>3	31 (44.3%)
Incidental	54 (76.1%)
Tumor size	4,1 (1,3 – 7,0)
**Histological subtype**	
Clear cells	34 (48%)
Others malignant	28 (39%)
Benign	9 (13%)
**Patological Stage**	
T1a	39 (55%)
T1b	21 (30%)
T2	6 (8%)
T3a	5 (7%)
**Margin status**	
Negative	67 (94%)
Positive	4 (6%)
**Surgical intervention**	
Laparoscopic partial nephrectomy	32 (45.1%)
Open partial nephrectomy	28 (39,4%)
Laparoscopic total nephrectomy	8 (11.3%)
Open total nephrectomy	3 (4.2%)
Pre-operative creatinine	0.9±0.18
Post-operative creatinine	1.0+0,24
Pre-operative hemoglobin	13.7±1.3
Post-operative hemoglobin	11,3±2,6

We analyzed intra operative outcomes (operative time - OT, warm ischemia time - WIT, estimated blood loss - EBL, conversion to open approach, conversion to total nephrectomy) and complicationrates recorded during the first 90 days after surgery and classified them according to the Clavien-Dindo classification system (13). The operative time was considered long when >180 minutes (14-16), WIT when >20 minutes (17-19) and EBL when ≥1000mL (20). The pathological margin status of the specimens was also analysed.

Further, we divided the results into groups according to the ASA (American Society of Anesthesiologists), Charlson comorbidity index (21) to verify the association of comorbidities on the results.

The R.E.N.A.L. score were tested for their ability to predict surgical outcomes and complications using receiver operating characteristic (ROC) curves. The overall performance of the ROC analysis was quantified by computing the area under the curve (AUC). An area of 1 indicated perfect performance, while 0.5 indicated a performance that was not different from a result that could have been obtained by chance. Using ROC analysis, the optimal sensitivity and specificity of R.E.N.A.L. were determined using various threshold values and Youden index method for the prediction of outcomes. Relative risk was calculated using Mantel-Haenszel analysis. The Fisher’s exact test and chi-square test were used to compare proportions. We also performed univariate Cox regression analysis to select variables that showed significant associations with the dependent variables. Only these were included in the multivariate Cox proportional-hazards model in a stepwise method. Two-tailed p <0.05 was considered to indicate statistical significance. The analyses were conducted using SPSS statistical software (version 17.0).

## RESULTS

Seventy-one patients were included in a intention to treat partial nephrectomy analysis. Clinical and pathological features are exposed in Table-1. No statistical difference was found in RNS groups regarding to age, BMI, ASA and Charlson score (Table-2).

**Table 2:** Distribution of variables according to renal score.

	LC	MC	HC	p
Lengh of stay (days)	3.6	3.9	4.2	ns
Operative Time (min)	134	163	185	<0.05 *
Estimated blood loss (mL)	376	460	347	ns
Warm isquemia time (min)	10	15	20	<0.05**
Clavien ≥3	0	7 (16.6%)	1 (4.5%)	ns
ASA >2	1(14.3%)	4 (9.5%)	1 (4.5%)	ns
BMI	27.7	27.6	27.6	ns
Charlson ≤3	6 (85.7%)	36 (85.7%)	18 (85.4%)	ns
Incidental	7 (100%)	33 (78.6%)	14 (63.6%)	0.05
Positive margins	1(14.3%)	2 (4.8%)	1 (4.5%)	0.17
Clear cell histology subtype	2 (28.6%)	17 (44.7%)	15 (68.2%)	0.5
LPN	5 (71.4%)	22(52.4%)	5(22.7%)	0.01

## SURGICAL OUTCOMES

### Conversion rate

**Figure 1:**
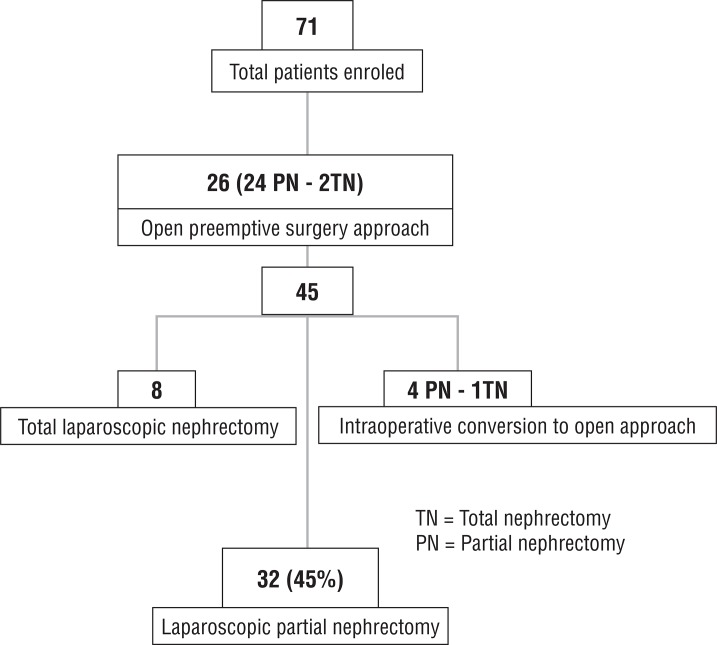
Conversion rate.

Of the 71 patients included, 26 had preemptive open conversion (2 total and 24 partial).Forty-five subjects initially underwent laparoscopic procedure, 8 were converted to laparoscopic total and 5 to open nephrectomy (4 partial and 1 total) (Figure-1).

**Figure 2:**
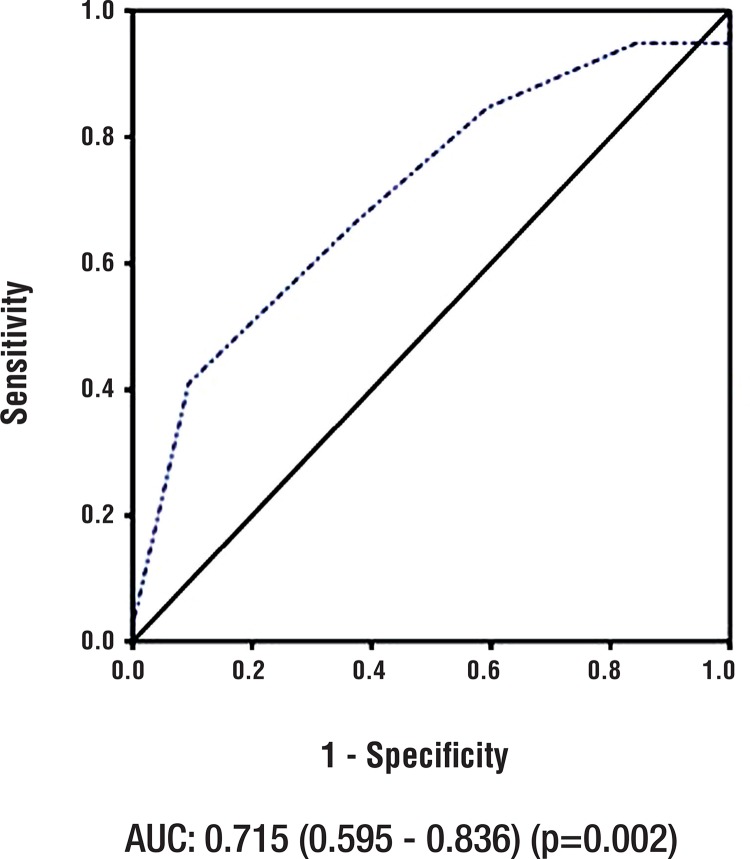
ROC curve: RENAL x conversion rate.

ROC curve was performed to test the accuracy of RNS to predict conversion rate. The AUC was 0.715 (0.595-0.836) (p=0.002) (Figure-2). The best specificity cut-off was RENAL ≥9. Patients with RNS<9 were most often subjected to PN (93% x 72%, p=0.03) and LPN (56.8% x 28%, p 0.02) (Table-3).

**Table 3:** Conversion rate according to RENAL ≥9.

RENAL	LPN	Others	p
<9	25 (54.3%)	21 (45.7%)	0.02
≥9	7 (28%)	18 (72%)	

Perioperative outcomes were not different in distinct surgical access. (Table-4)

**Table 4:** Perioperative outcomes and surgical approach.

	LPN	OPN		LTN	OTN	p
OT	174±51 (90-300)	155±50 (70-261)		189±77 (120-354)	133±38 (90-160)	NS
WIT	17 (10-35)	11.5 (8-30)		-	-	NS
EBL	376 (223 -529)	491 (318-662)		293 (123-465)	513 (-/1597)	NS
LOS	3.6+1.1 (1-6)	4.3+1.2 (3/7)		4.1+2.2 (2/9)	3.7+1.2 (3-5)	NS
Clavien-Dindo ≥3	3 (9.4%)	3 (10.7%)		2 (25%)		

**OT =** Operative time - mean (95%IC)

**WIT =** Warm ischemia time - median (95%IC)

**EBL =** Estimated blood loss - mean (95%IC)

**LOS =** Length of stay - mean (min - max)

**LPN =** Laparoscopic partial nephrectomy

**OPN =** Open partial nephrectomy

**LTN =** Laparoscopic total nephrectomy

**OTN =** Open total nephrectomy

### Warm ischemia time

The median duration of ischemia increased with tumors anatomical complexities, according to the RNS (LC: 10 minutes; MC: 15 minutes; HC: 20 minutes - p<0.01). There were no patients in LC group with WIT ≥20min (LC 0xMC 41.4% x HC 64.3%, p=0.03)

We performed a ROC curve analysis and found an AUC=0.598, which was not significant (p=0.252); this finding demonstrates that there is no cutoff score of RNS that is able to predict a clamping time <20 or ≥20.

### Operative time

The mean operative time was longer in HC (185 min) than in MC (163 min) or LC (134 min)p<0.05. The ROC curve demonstrated that the RNS could predict a prolonged surgery time, with an area under the curve of 0.63 (p=0.05) (Graph-1). Using the Youden index to predict the best cutoff point, we found R.E.N.A.L. ≥8 as a predictor of surgical time ≥180 minutes, with a sensitivity of 89.3% and specificity of 37.2%. The odds of having surgery time >180 min. was 4.94 times greater in patients with a score ≥8 (p=0.020).

### Estimated blood loss

The average EBL in the groups were, respectively, 376, 460 and 347mL for LC, MC, HC. These values lacked both clinical and statistical difference. Seven patients had ≥1000mLof bleeding and 1 was transfused (2000mL of bleeding). There were no association with R.E.N.A.L. complexity groups: 1LC; 5MC; 1HC.

### Margin status

No TN patients had positive surgical margins, although 4PN patients (4/60) had positive surgical margins, 3LPN and 1 OPN.

### Post-operative complications

No patients presented with post-operative bleeding, urinary fistulas, pseudoaneurysms with clinical symptoms or deaths during the follow-up time. Eight (11.3%) patients had major complications (Clavien≥3). However, none of these complications were observed in LC group, instead occurring in 7 (16.6%) and 1 (4.5%) individuals in MC and HC groups, respectively.

In the logistic regression analysis,RNS, surgical approach (open or laparoscopic) and operative time were not related to Clavien ≥3 (Table-5).

**Table 5:** Predictors of Clavien≥3.

	OR	95% CI	p
RENAL	0.95	0.6-1.5	0.84
RENAL-L	0.76	0.5-1.2	0.82
ASA	11.48	1.9-69.5	0.008
Age ≥65	6.45	1.2-24.8	0.043
Surgical approach	0.9	0.8-1.3	0.783
Operative time	1.01	0.99-1.0	0.1

We found that the addition of each unit to the ASA score increased the chance of having Clavien ≥3 by 11.48 times (p=0.008). The ROC curve analysis got an AUC of 0.69 (p=0.084). The best number given by the Youden index was 3, with a sensitivity of 37.5% and specificity of 95.2%. From this value, we applied logistic regression with ASA categorizations, and the odds of having Clavien ≥3 were 12 times greater for individuals with an ASA >3 (95%CI=2-69; p=0.008).

Furthermore, age was significantly associated with major complications. We found that each additionalyear of age increased the chance of having Clavien ≥3 by 1.08 times (p=0.043). The AUC for this was 0.72 (p=0.043), demonstrating that age is a predictive variable for Clavien ≥3. Searching for the best cutoff according to the Youden method, we found that age ≥66 years had a sensitivity of 75.0% and a specificity of 68.3%. From this value, we applied logistic regression with age categorization and found that those with age ≥66 were 6.45 times more likely to have Clavien ≥3 (95%CI=1.2-34.8; p=0.030).

## DISCUSSION

The majority of papers that have studied nephrometry score systems are retrospective. Thus, confounding factors are usually adjusted for in the statistical analysis. On the other hand, in our study, we adjusted for these factors within the methodology, performing the study prospectively and excluding patients with anatomical features that could interfere with the perioperative results. These stringent inclusion criteria led to a significant loss of sample size, which could have reduced the power of our analysis. In the present study, we evaluate the accuracy of R.E.N.A.L. nephrometry system in predicting outcomes in partial nephrectomies for <7cm kidney cancers because we don’t have literature to support routine elective partial nephrectomy in >7cm tumors.

From a technical point of view, the choice between partial or total nephrectomy is still very subjective and even experienced surgeons often are in doubt whether the tumor can be extirpated in order to preserve functional renal parenchyma and in a minimally invasive approach.With nephrometry scores using, one can obtain objective parameters to predict conversion rates.In our sample, we can identify patients with greater chances of conversion. Individuals with RNS≥9 are at high risk, and perhaps would be better approached by open surgery.

Funahashi et al. (22) retrospectively evaluated anatomical data of renal tumor associated with the access route to partial nephrectomy and found that the tumor’s relationship to the renal surface (endophytic character) and the distance from the renal sinus affected the surgeon’s decision to open access route or minimally invasive, and tumor size did not influence that decision.Gill et al. (23) in a similar analysis with 771 LPN and 1029 OPN reported that tumor size (2.6cm LPN vs. 3.3cm OPN) and endophytic character (34.4% LPN vs. 53.3% OPN) were significantly different between the two access routes (17).Naya et al. (8) evaluated factors that influence the frequency of LTN (68 patients) vs. LPN (74 patients). They found that the RNS up to 8 was the best cut off for patients selection for LPN.As these data are being validated by larger studies, it will allow better predict the chances of conversion, improving the anesthetic and surgical planning and patient preparation for this possibility. Moreover, technically favorable tumors should be most operated by LPN.

A systematic review from American Society of Anesthesiologists defines as prolonged surgery intervals from 2.5 hours to 4 hours (14). Also others references confirm these information based on increasing post operative complications (15, 16). Ng et al. (24) reported an OT of 3.5 hours in LPN. Marszalek et al. (25) had an average time of 139 min. We had a mean OT of 174 minutes in LPN and RNS was significantly associated with a prolonged surgery time. Data showed that RNS≥8 was a predictor of surgical time ≥180 minutes, with a sensitivity of 89.3%. This indicates that, in cases of a CT scan showing a renal mass with RNS <8, the surgeon could predict that rarely OT will exceed 3 hours.

Although controversial in literature, several clinical studies suggest that the maximum period of WIT time for preservation of renal function should not exceed 20min (17, 19, 26). Previous studies have reported differences in WIT among R.E.N.A.L. groups (9, 10). In our series, we found that WIT was statistically greater in the high complexity group. However, the difference was not clinically significant, as no group had a median WIT greater than 20 minutes. Advancing this analysis, we found that there were no cutoff scores of RENAL able to predict a clamping time <20 or ≥20 min. It is likely that the low mean WIT in our patients contributed to these results. To reduce WIT, our group unclamps the kidney vessels early, immediately after the first external parenchymal suture has been placed. Otherwise some studies have demonstrated a correlation between RNS and WIT. Hayn et al. (27) found, in a series with 141 laparoscopic partial nephrectomies, WITs of 16, 23 and 31 minutes for the low, medium and high complexity groups, p<0.001.

The hemorrhagic shock scale proposed by ATLS (Advanced Trauma Life Support) consider bloodloss between 15-30% of body volume or 750 to 1500mL (Class II) a significant clinical bleeding, because patients experiment tachycardia, tachypnea and elevates plasma levels of catecholamins (20).RNS was not good predictors of EBL. In our series and in a recently published paper, intraoperative bleeding was not clinically different among RNS groups (LC: 135mL; MC: 210mL; HC: 314mL) (11). These results could be explained by a good intra-operative vascular control of the hilum and a good suture repair of the kidney parenchyma, achieved using open, laparoscopic or robotic techniques.

The overall incidence of urological complications after LPN has been reported at 9.0% (12). Simmons and Gill (28) found no correlation between tumor size and centrality with the incidence of complications after LPN, on either the univariate or multivariate analyses. According to a recently published paper (12), a higher RNS was significantly associated with an increased incidence of Clavien grade III. In our series, RNS did not show any relation to post-operative complications. On the other hand, the ASA score and patient’s age were significantly associated with Clavien ≥3. In our sample, baseline patient characteristics were more important that anatomical tumor characteristics in predicting complications.

Turna et al. (29) reported a 2.4% incidence of postoperative urinary fistula after LPN, but no independent predictors of this outcome were found. Recently, another group (30) reported that each unit of increase in RNS was associated with an increased likelihood of a postoperative urine leak. We did not encounter any urinary leaks postoperatively, and no patients had to use a ureteral catheter. A first line suture performed with 2.0 Vicryl SH needle in all patients with a deep defect in the renal parenchyma may be sufficient to include the collecting system effectively.

From now, we have in literature 10 scores that use anatomic features involved in complexity of partial nephrectomies. Although some studies have been showing association between them and surgical outcomes, no one have accuracy strongly tested and validated (31).

## CONCLUSIONS

There is a growing need to objectively measure the complexity of kidney tumors due to the use of minimally invasive procedures that require greater operative skill. In respect to ≤7cm tumors,R.E.N.A.L. score, in this data, was a good method in predicting surgical access route and type of nephrectomy. Also was associated with OT and WIT, but with weak accuracy. On the other hand, RNS was not associated with Clavien >3, EBL, LOS or positive surgical margin. These observations must be tested by other groups in a major population.

## ABBREVIATIONS

ASA: American Society of Anesthesiologists
AUC: area under the curve
BMI: body mass index
EBL: estimated blood loss
HC: high complexity
LC: low complexity
LOS =: length of stay
LPN =: laparoscopic partial nephrectomy
MC: *medium complexity*
OPN: open partial nephrectomy
OT: operative time
PN =: partial nephrectomy
RNS: R.E.N.A.L. nephrometry system
ROC curve: receiver operating characteristic curve
RTs =: Renal Tumors
TN: total nephrectomy
WIT: warm ischemia time
